# The Use of Nonmonetary Outcomes in Health-Related Delay Discounting Research: Review and Recommendations

**DOI:** 10.1007/s40614-024-00403-7

**Published:** 2024-04-29

**Authors:** Erin B. Rasmussen, Lillith Camp, Steven R. Lawyer

**Affiliations:** Department of Psychology, Idaho State University, Stop 8112, Pocatello, ID 83209-8112 USA

**Keywords:** Delay discounting, Domain effects, Nonmonetary outcomes, Obesity, Reinforcer pathology, Substance use disorders

## Abstract

Delay discounting (DD) refers to the tendency to devalue an outcome as a function of its delay. Most contemporary human DD research uses hypothetical money to assess individual rates of DD. However, nonmonetary outcomes such as food, substances of misuse, and sexual outcomes have been used as well, and have advantages because of their connections to health. This article reviews the literature on the use of nonmonetary outcomes of food, drugs, and sexual outcomes in relation to health and reinforcer pathologies such as substance use disorders, obesity, and sexual risk behaviors, respectively, and makes a case for their use in discounting research. First, food, substances, and sex may be more ecologically valid outcomes than money in terms of their connections to health problems and reinforcer pathologies. Second, consistent trends in commodity-specific (i.e., domain) effects, in which nonmonetary outcomes are discounted more steeply than money, enhance variation in discounting values. Third, commodity-specific changes in discounting with treatments designed to change health choices are described. Finally, methodological trends such as test–retest reliability, magnitude effects, the use of hypothetical versus real outcomes, and age-related effects are discussed in relation to the three outcome types and compared to trends with monetary discounting. Limitations that center around individual preferences, nonsystematic data, and deprivation are discussed. We argue that researchers can enhance their DD research, especially those related to health problems and reinforcer pathologies, with the use of nonmonetary outcomes. Recommendations for future directions of research are delineated.

Delay discounting (DD) refers to individual devaluation a reward as a function of its delay to receipt (Ainslie, 1975; Rachlin et al., 1991). Originally referred to as a form of “impulsivity,” research on temporal sensitivity to reward has its roots with the “marshmallow task,” or preschool-aged children’s single choice between a small amount of food (e.g., a single marshmallow or cookie) now, or more food after a delay (Mischel & Ebbesen, 1970). Performance on this task, especially difficulties in delaying gratification to the larger, delayed food option, predicted a large number of outcomes, including school success (Shoda et al., 1990), later SAT scores (Shoda et al., 1990), academic achievement (Watts et al., 2018), adolescent social and cognitive function (Mischel et al., 1988), and even later obesity (Schlam et al., 2013). It should be noted that most of these later outcomes, except for obesity, were not related to marshmallows or food at all. This cross-commodity pattern, however, likely set the stage for considering discounting as a general behavioral process that underlies a wide range of temporally based decisions that vary across a diverse array of outcome types (see Odum et al., 2020, for review).

To date, the research on DD has come a long way since Mischel’s original studies and has generated a vast database that includes refinements in procedures such as assessing preferences for not just one choice, but across *a series of* choices between smaller, sooner outcomes versus larger, later outcomes. This procedural detail alone allows a fuller characterization of choice patterns and preferences across varying amounts of an outcome and delays to those outcomes. There are also varying methodological details in choice presentations, such as the use of real outcomes versus those that are hypothetical (e.g., Johnson & Bickel, 2002; Lagorio & Madden, 2005; Lawyer et al., 2011, 2022; Madden et al., 2004; Robertson & Rasmussen, 2018; cf. Hinvest & Anderson, 2010), variations in magnitude (e.g., Baker et al., 2003; Green et al., 2013; Mellis et al. 2017), the method of presentation of choices (e.g., pencil-paper vs. computerized, choice questionnaires; Kirby & Maraković, 1996; Hendrickson et al., 2015), and presentation sequence of choices (e.g., random vs. ordered sequence; Robles & Vargas, 2007), just to name a few. Research also includes the use of nonhuman animals, including rodents, pigeons and others, which assists with identifying neural substrates and allele patterns that underlie discounting processes (see LeComte & Rasmussen, 2024; Vanderveldt et al., 2016 for reviews; Perry et al., 2007; Robertson & Rasmussen, 2017).

Another methodological detail in discounting that has evolved since Mischel’s marshmallow task is the wide range of outcomes that are used in choices. Indeed, the most frequently used commodity in contemporary DD studies is hypothetical money. The use of monetary outcomes has benefits that will later be described in this article, and there is certainly a place for their use. However, the point of this article is to draw attention to the underrepresented use of nonmonetary outcomes in DD studies with humans. We believe there are excellent reasons for the use of nonmonetary outcomes in discounting studies, especially in the context of health-related phenomena, such as substance use disorders, obesity, and sexual risk taking. We wish to make a case for their more frequent use in human research. As such, we will primarily describe DD research with humans, though occasionally animal studies will also be cited.

## The Delay Discounting Paradigm

**Fig. 1:**
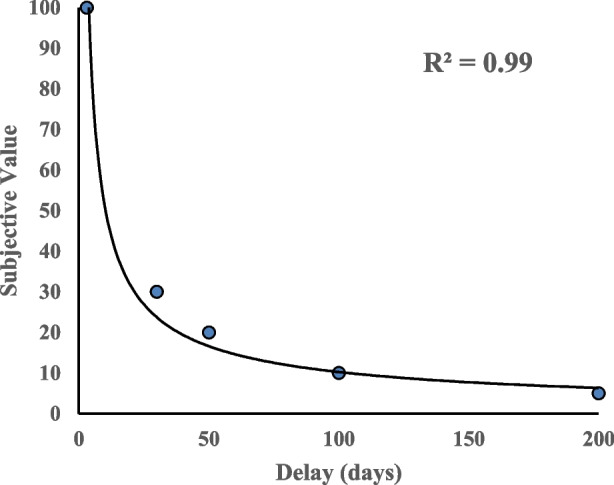
A typical delay discounting curve with hypothetical money

In a typical DD paradigm with humans, individuals make choices between a series of smaller, immediate hypothetical monetary outcomes versus those that are larger, and delayed. For example, one choice question may be: “Which would you prefer? $100 now OR $100 dollars in 30 days?” When presented with this type of choice, participants generally choose the more immediate reward of $100 now in this scenario, as the delayed $100 is devalued by time to its receipt. After each choice, a similar subsequent choice is given in which one of the amounts is altered to approximate the value of the other. For instance, the smaller, sooner option may be systematically decreased to approximate the value of the larger, later option: “Which would you prefer? $90 now OR $100 dollars in 30 days?” The systematic decrease of the smaller amount continues with subsequent choices until a preference reversal occurs (i.e., the participant switches preference to the larger, delayed outcome). From there an indifference point between the two outcomes is established by finding the median of the values that flank the preference reversal. This indicates the current subjective value of the larger outcome after that delay. This procedure is repeated across a series of additional delays (e.g., 50–365 days). Indifference points are plotted against these delays and a DD curve is established (see Fig. 1). The subjective value of the delayed outcome tends to decrease hyperbolically as the delay to the large outcome increases and has been characterized most commonly by Mazur’s one-parameter hyperbolic decay function (Mazur, 1987) or by one of two 2-parameter models, which incorporates a scaling variable (Green & Myerson, 2004; Rachlin, 2006).

Differences in sensitivity to delay are indicated by the rate of decline in the subjective value of the delayed outcome. This rate is associated with patterns of preference for smaller–sooner outcomes (high discounting rates) over the larger–delayed outcome across the series of choices. Steep patterns of DD are associated with a variety of health and mental health concerns that often involve challenges in self-regulatory behavior, including substance use disorders (Bornovalova et al., 2005; Kirby et al., 1999; Madden et al., 1997; Petry, 2001), obesity (Rasmussen et al., 2010; Saelens & Epstein, 1996), binge disorder (Manwaring et al., 2011; Steward et al., 2017) sexual risk taking (Chesson et al., 2006; Johnson & Bruner, 2012; Lawyer & Mahoney, 2017), indoor tanning (Sheffer et al., 2018), and gambling (Reynolds et al., 2006).

DD as a preference for immediate outcomes has historically been viewed as one of various behavioral indicators of the construct impulsivity (e.g., Ainslie, 1975; De Wit, 2008; Evenden, 1999). However, recent research (see Strickland & Johnson, 2020) examining the validity and utility of the construct of impulsivity suggests that DD is a unique and independent process and should be treated as separate from other putative measures of impulsivity, e.g., response inhibition and riskiness. Moreover, the term “impulsive” is often used pejoratively because of its association with health problems such as substance use disorders. The assumption is that impulsive behavior is problematic, even though there could be conditions in which a pattern of choice for smaller, sooner outcomes may have survival value (such as when resources are scarce; see Rodriguez et al., 2021) and therefore, may be a rational and appropriate choice. Therefore, there are good reasons to disassociate the process of discounting from impulsivity.

In any case, there is clear evidence that DD represents a variable consistent with contemporary personality theories (Mischel & Shoda, 1995; Odum, 2011a). DD patterns are relatively consistent across time (Beck & Triplett, 2009) and are relatively similar across commodities, suggesting a more general process consistent with a trait (Odum, 2011a, b; Odum et al., 2020). For example, Odum (2011a) conducted an archival data analysis of DD studies that examined patterns of DD across more than one commodity and found that although patterns of DD differed across commodities (e.g., money vs. food), the overall patterns were nonetheless consistently correlated (steep discounters for one commodity tend to be steep discounters for another), suggesting that patterns of DD are similar across commodity type.

Despite these intercommodity correlations that support discounting as a trait variable, discounting can still be altered as a state variable. Rung and Madden’s (2018) systematic review found that DD is sensitive to situation-specific influences, such as episodic future thinking and mindfulness-based interventions. However, the effect sizes of manipulations of discounting are generally quite small—usually less than 0.2. Therefore, although discounting is viewed as a trait-like pattern of behavior, variables can shift patterns in smaller, more nuanced directions, which may contribute to probabilistic shifts towards, or away from, self-regulatory problems.

## Delay Discounting with Monetary Outcomes

As mentioned, hypothetical money, by far, is the most frequently used outcome in DD research (Madden & Bickel, 2010). There are compelling reasons for this. For instance, the value of currency is easy to quantify and is easily understood. Most people can identify and understand what a particular amount of the currency in their own country can buy. Money is also a conditioned and generalizable reinforcer, in that it has been paired with a variety of primary reinforcers in a manner that allows it to hold a reinforcing function across a variety of settings and contexts (e.g., Williams, 1994a, 1994b). Because currency is part of most cultures, people who have experience with exchanging money for goods also find money to be reinforcing. Its value may be scaled slightly from person to person, with variables like income—e.g., compare the value of $100 to a college student on a tighter budget versus someone in the upper 1% of income—but both individuals likely behave as though the $100 has value. In addition, the use of money (or points exchangeable for money) as a reinforcer has historically been one of the most frequently used stimuli in human operant laboratory research (see Galizio & Buskist, 1988; Kollins et al., 1997), as opposed to primary reinforcers such as food, which are typically used in operant studies using animals. Money is also used as an incentive to participate in research, which also attests to its potency (Rudy et al., 1994; Stunkel & Grady, 2011).

Because money is convenient to use in discounting research, it is not surprising to see its use in research questions related to health. Perhaps the most well-researched health area to which discounting has been applied is substance misuse and substance use disorders. A rich literature compares monetary DD between individuals who chronically use substances or those with substance use disorders versus those who do not. The findings consistently show that the sample with a substance use history discounts money more steeply than controls (see reviews and meta-analyses by Amlung et al., 2017; MacKillop et al., 2011; Weinsztok et al., 2021; Yi et al., 2010). For instance, those who smoke or vape nicotine regularly discount money more than controls who do not use, or formerly use, nicotine (e.g., Johnson et al., 2007). Those who drink alcohol heavily tend to discount money more than those who do not drink alcohol (e.g., Field et al., 2007; Vuchinich & Simpson, 1998). This pattern is also shown with individuals who have substance use disorders with illicit compounds such as cocaine (Heil et al., 2006), heroin, or other opioids (e.g., Madden et al., 1999)—monetary DD is steeper with these populations compared to controls with no or low-risk patterns of use.

Another health area to which monetary discounting has been applied has been obesity. Weller at al. (2008) first showed that women, though not men, with obesity discounted money more steeply than healthy-weight controls. Other studies have replicated that effect (e.g., Appelhans et al., 2012; Field et al., 2011; Jarmolowicz et al., 2014a, 2014b). Indeed, a meta-analysis by Amlung et al. (2016) reported a moderate relation between obesity and monetary discounting across studies. It should be noted, though, that some studies have reported no association between monetary discounting and obesity (see meta-analysis by Tang et al., 2019). The authors note that studies that did not use “best practice methods,” such as using appropriate statistical analyses and using a real payoff for some of the choices, tended to be the ones that did not find this relation. (As for the latter issue, it is unclear that using real or potentially real outcomes is a “best practice” measure since to date no differences in discounting have been observed between conditions that use hypothetical vs real and potentially real monetary outcomes, and in some cases, have been shown to be statistically equivalent; Lawyer et al., 2011; Lagorio & Madden, 2005; Madden et al., 2003, 2004; Matusiewicz et al., 2013). Nonetheless, the research trend is that steeper monetary discounting is associated with obesity.

It is important to note that monetary discounting has also been used in other contexts, such as gambling (see Reynolds, 2006), money mismanagement (Hamilton et al., 2012), and with those who seek payday and title loans (Mahoney & Lawyer, 2016). In all instances, these potential debt problems involve choice patterns for immediate money over larger, later monetary consequences. Therefore, monetary outcomes would seem to be an ecologically valid outcome to use in these discounting studies. But when it comes to choices that are relevant to health problems, such as substance use disorders or obesity, it may be more relevant and ecologically valid to examine discounting with outcomes other than money, especially outcomes that are *related directly* to choices that lead to these health problems.

This is indeed the purpose of this article. We wish to review discounting research on the use of the specific nonmonetary outcomes of food, drugs, and sex. There are important reasons for discussing DD beyond the use of monetary outcomes. First, these nonmonetary outcomes are ecologically valid as they are directly embedded in the choices and consumption of reinforcers related to specific health problems, such as substance use, obesity, and sexual risk taking, respectively. Second, these nonmonetary outcomes are discounted more steeply than monetary outcomes (called commodity-specific effects), creating a larger window of variability in examining the potential for interventions or variables that may change discounting in the context of such health choices. Third, some studies that evaluate health-related treatments with monetary and nonmonetary outcomes show commodity-specific changes in discounting that relate to the outcome associated with the health problem (and not money); therefore, some treatments may be more sensitive to change with nonmonetary outcomes. Finally, discounting research with nonmonetary outcomes shows some methodological trends in validity and reliability that are akin to those shown with monetary outcomes. We will describe these literature trends in subsequent sections. To address the first issue of ecological validity, we begin with a theoretical framework that covers a range of health behaviors that are related to the high-risk, immediate consumption of reinforcers—the reinforcer pathology model.

## Ecological Validity: Delay Discounting and the Reinforcer Pathology Model

In some instances, the preferences or hedonic value for certain stimuli or outcomes may be so strong that reinforcer pathologies (Bickel & Jarmolowicz, 2011; Bickel et al., 2014) may develop. The term reinforcer pathology refers to a pattern of overconsumption of a reinforcer, such as drugs or food, in which two behavioral processes are involved (Bickel et al., 2011a, b, 2014). One process involves overvaluation of a reinforcer by way of excessive demand for the reinforcer, which is measured as insensitivity to effort that produces access to the reinforcer (also known as inelasticity). In other words, an organism will continue to defend their consumption of a reinforcer, despite effort-based (on monetary) increases in price. The second process is steep DD, especially for the reinforcer of interest. The term “reinforcer pathology” is a more useful term than addiction, which is fraught with overuse and has causal status for overconsumption of a reinforcer that may interfere with functioning in other aspects of life. Placing the focus on these processes helps researchers concentrate efforts on changing the behavioral processes (i.e., the contextual relation between the behavior and the reinforcer) that underlie the problem behavior instead of changing the hypothetical construct of addiction.

The reinforcer pathology model has been applied to substance use disorders for illicit compounds (e.g., Bickel et al., 2011a, b, 2014, 2017), alcohol misuse (Lemley et al., 2016; Minhas et al., 2020; Stancato et al., 2020), and cigarette smoking (Garcia-Pérez et al., 2021; Weidberg et al., 2019). In addition, the model has been applied to obesity (DeHart et al., 2020; Deshpande et al., 2019), sexual risk-taking behavior, such as not wearing condoms during sex (Harsin et al., 2021), and overconsumption of indoor tanning (Reed, 2015).

The process of DD, although only half of the reinforcer pathology model, is the focus of this article, but it is noteworthy that a large proportion of the literature on discounting in and of itself focuses on health-related problems in special populations that have specific reinforcer pathologies, such as substance use disorders (SUDs; Bickel et al., 2012, 2014; see MacKillop et al., 2011, for review) and obesity (e.g., see reviews by Amlung et al., 2016, and Epstein et al., 2010; see also Tang et al., 2019). Indeed, DD is considered a trans-disease process that underlies choices involved in these types of health problems (Bickel & Mueller, 2009). It is interesting that money is often used as the outcome in these studies to characterize the differences in discounting between those with specific reinforcer pathologies versus controls (e.g., Weller et al., 2008). Though there are some studies that use a specific drug or food in discounting studies that is relevant to the reinforcer pathology of interest (e.g., Madden et al., 1999; Rasmussen et al., 2010), these are fewer than the base rate. This trend in the literature draws attention, because using a “proxy” outcome like money takes researchers one step away from understanding choices for outcomes that are *directly involved* in a reinforcer pathology. In other words, understanding choices about drugs that a sample of individuals with SUDs is more direct, ecologically valid. And may be more amenable to treatment than their monetary choices.

Food, substances (drugs), and sexual outcomes are three types of nonmonetary outcomes that are hyperbolically discounted and are likely involved in reinforcer pathologies. We will therefore describe research on each outcome type, including research trends that that each is more strongly discounted than money (i.e., commodity-specific effects). We then will characterize their associations with reinforcer pathologies, with attention to treatment studies in which changes in discounting for nonmonetary versus monetary outcomes are compared. The majority of these studies were conducted on adult humans, except where noted.

## Discounting with Nonmonetary Outcomes, Related Health Problems, and Commodity-Specific Effects

### Delay Discounting for Food

Food is the most studied nonmonetary outcome in human DD research; there are valid reasons for that. Everyone must eat to survive. The value of food is present at birth (i.e., innate or unlearned) and is therefore considered a primary reinforcer. Human studies with both real and hypothetical food outcomes show that food is hyperbolically discounted (e.g., Kirk & Logue, 1997; Odum et al., 2006; Odum & Rainaud, 2003; Rasmussen et al., 2010).

DD for nonmonetary outcomes such as food often is compared to monetary discounting as a method of validation. It should be noted that to directly compare nonmonetary outcomes to monetary outcomes, their values must first be equated. Studies that equate or standardize these values often reveal consistent commodity-specific (also called domain) effects. One such effect is that food is discounted more steeply than money (see review by Odum et al., 2020). For instance, Odum and Rainaud (2003) compared discounting for monetary, dollar-equivalent amounts of pizza- and alcohol-related outcomes and found that money was discounted less steeply than food and alcohol, which were discounted similarly. Odum et al. (2006) replicated these findings with money and food and extended them by examining magnitude effects, in that smaller amounts of food were discounted more steeply than larger amounts. Estle et al. (2007) also compared discounting for hypothetical money to beer, candy, and soda, and found that beer, candy, and soda were discounted similarly, but when compared to money, all three outcomes were discounted more steeply than money. The commodity effect with food versus money has been replicated in other studies as well (e.g., Charlton & Fantino, 2008; Holt et al., 2014).

Other studies show commodity-specific effects even among different food-related commodities. In a study by Tsukayama and Duckworth (2010), participants rated the hedonics (individual level of temptation from 1 to 5 with 5 being the most tempting) of beer, candy, and chips and then completed DD tasks for each commodity. They found that DD for each food type increased with hedonic rating. The authors also compared discounting for beer and candy among beer lovers (those who rated beer as a 5) and candy lovers (those who rated candy as a 5). Although beer lovers showed overall higher discounting than candy lovers, when discounting for beer only was examined, beer lovers exhibited significantly steeper discounting for beer than candy lovers. The results illustrate that even within the domain of food, discounting for specific kinds of food can strongly relate to preferences or hedonic value ratings.

There are a number of reasons why food is likely to be more steeply discounted than money. One may have to do with the nature of food as a primary reinforcer and money as a secondary reinforcer. But, simply stating that primary reinforcers are more steeply discounted than secondary reinforcers may not be a satisfying explanation and unpacking the properties of this difference may lead to a better understanding. For example, food is immediately consumable, and money is not. Studies that vary the degree to which the outcome is immediately consumable have shown that this quality is important. For example, Charlton and Fantino (2008) compared discounting among commodities that were immediately consumable, but also varied in metabolic properties. Music, books, and CDs, all of which are immediately consumable, but not metabolic, were compared to money and food discounting. Music, books, and CDs were discounted more steeply than money, but food was discounted the steepest, suggesting that both the qualities of immediate consumption and metabolic nature contribute to steeper discounting.

Perishability, or the putative shelf life of an outcome, may also play a role in commodity-specific effects with discounting. Holt et al. (2014) compared discounting between food, money, and sexual outcomes. The authors argued that though sex and food are both primary reinforcers, food is perishable, whereas sex is not (although the availability and desire for sex may be variable), so there may be differences in their discounting rates. Indeed, of the three outcomes, money was discounted least steeply, followed by sex. Food was the most steeply discounted outcome and was significantly steeper than sex. Therefore, perishability likely contributes to commodity-specific effects observed in food and other nonmonetary outcomes.

Fungibility, or the degree to which a commodity is exchangeable for other goods, may also be a property that plays a role in commodity-specific effects in food discounting. Holt et al. (2016) compared DD for a variety of commodities that varied in their fungibility. Items that were highly fungible, such as money, Visa gift cards, and grocery gift cards were less steeply discounted than food such as candy and pizza slices and even nonfood items such as jeans and pizza gift cards. It is important to note that items that were most perishable, but least fungible were discounted the most steeply. For example, of pizza slices and jeans, both of which have low fungibility, pizza was discounted more steeply than jeans. Candy, though, likely has lower perishability than pizza, and both of these commodities were discounted similarly and steeply. Regardless, the properties of low fungibility and high perishability tend to predict steeper discounting, which may explain also why food is a highly discounted commodity.

### Food Discounting and Reinforcer Pathologies

For some individuals, food has high valuation that can develop into a significant health problem such as obesity (Epstein et al., 2000; Epstein et al., 2010; Rasmussen et al., 2010) Obesity increases the risk for chronic health problems, such as type-2 diabetes, heart disease, liver disease, and some types of cancer (Centers for Disease Control & Prevention [CDC], 2023b). At present, over 42% of Americans are obese, and an additional 36% are overweight (CDC, 2023a). A higher prevalence of obesity has been observed in industrialized nations (e.g., Doak & Popkin, 2001). Individuals with obesity show steeper DD compared to controls, and some of this research indeed has been conducted with monetary outcomes (see Amlung et al., 2016; Jarmolowicz et al., 2017; Weller et al., 2008). It is important to note that obesity-related discounting has also been observed with food discounting, a seemingly more ecologically valid outcome that is directly related to food choices that may lead to obesity (Rasmussen et al., 2010; Hendrickson & Rasmussen, 2017; Hendrickson et al., 2015; Rodriguez et al., 2021). In these studies, participants were asked to imagine a 1-in cube as a bite of their favorite food and then made a series of choices between smaller, sooner vs larger, delayed bites of food, such as “would you rather have 3 bites of your favorite food now or 10 bites after 3 hours?” The range of bites represented those in a typical single sitting of eating a meal (1–10 bites on the lower end and up to 25 bites on the higher end) and the delay ranges (1–20 hr) were designed to reflect day-to-day food decisions. Participants were asked to not eat for at least 2 hr before sessions and in some cases, this is verified by a blood glucose test (e.g., Hendrickson & Rasmussen, 2017; Rasmussen et al., 2022; Rodriguez et al., 2021).

Results from these studies were consistent. Using a computerized-adjusting amount procedure, Rasmussen et al. (2010) first reported that individuals in the highest quartile of percent body fat (PBF) exhibited steeper discounting for bites of food than those in the lowest PBF quartile but there were no differences in monetary discounting. This obesity-related effect was replicated in some additional studies, including with using the Food Choice Questionnaire (FCQ; Hendrickson & Rasmussen, 2013, 2017; Hendrickson et al., 2015; Rodriguez et al., 2021). The FCQ is a 27-item food choice questionnaire similar to the well-established Monetary Choice Questionnaire (MCQ) by Kirby and Maraković (1996). Across all of these studies, a PBF effect was found with food discounting, but never with monetary discounting.

Stress and sleep quality also differentially predict DD for food, but not for money. Undergraduate students are a population known to have demonstrably high stress levels and poor sleep quality (see review by Lyzwinski et al., 2018; Coughlin & Smith, 2014; Rahe et al., 2015; Sa et al., 2020), as well as weight gain during college (see meta-analysis Vella-Zarb & Elgar, 2009)—factors that are metabolically related. A study by Law and Rasmussen (2023) showed that higher stress levels in undergraduate students, which corresponds to stress-eating, exhibited steep discounting for food, but not money. In addition, poor sleep quality, a pattern with overeating at night, predicted greater preferences for larger, later, food-related, but not monetary, outcomes. Therefore, stress and sleep quality predicted two independent food-related discounting processes that are specifically related to metabolic processes. Because stress and sleep quality did not predict monetary discounting, it supports commodity-specificity to food discounting as a metabolic behavioral process.

### Food Delay Discounting and Treatment Specificity

Treatment effects on discounting processes that are specific to food are illustrated by some notable studies in the literature. Hendrickson and Rasmussen (2013) investigated the extent to which a mindful eating exercise influenced discounting. College students completed DD tasks for hypothetical food and monetary outcomes in a baseline session. In a second session, participants were randomly assigned to one of two conditions: a mindful eating condition or a control DVD condition. In the mindful eating exercise condition, participants were given four different bite-sized foods and instructed to eat them in a manner in which they objectively described properties of the food, such as sensory properties and physiological responses to the food, such as salivation. Participants in the control DVD condition watched a 50-min DVD that presented general nutrition information. They were also given the same foods, but they were not given instructions on how to consume them. Participants then repeated the food and monetary DD tasks. Results showed that those in the mindful eating condition had lower rates of discounting for hypothetical food compared to their baselines; there were no significant differences in DD in the control DVD condition. It is important to note that there were no differences in monetary discounting for either group.

Hendrickson and Rasmussen (2017) replicated and extended this procedure by including adolescents and adults, using the FCQ and MCQ as measures of food and money DD, respectively, and using an additional control condition. Similar to the 2013 study, participants assigned to the mindful eating condition discounted food, but not monetary outcomes, less steeply postsession. Neither control condition resulted in altered food or money discounting. Taken together, these findings support that mindful eating effects were commodity (food) specific. Treatments that specifically target food as an excessively valued or steeply discounted outcome may be successful at ameliorating reinforcer pathologies related to food.

### Summary

Food is a hyperbolically discounted outcome. Research supports commodity-specific (i.e., domain) effects, in which food is consistently discounted more steeply than money. This is likely because food is a primary reinforcer, is immediately consumable, metabolic, perishable, has lower fungibility, and perhaps higher lifetime experience compared to money. Because food as an outcome is more steeply discounted than money, a larger window of variability in data can be generated than simply using money alone. This range of variability may be ideal for research studies that require a wider distribution of data, such as those examining discounting processes in those with reinforcer pathologies with food. Indeed, steep food discounting has been shown in those with obesity, a reinforcer pathology related to food overconsumption. In addition, food discounting, and not monetary discounting, has been shown to be more sensitive to food-related state variables such as mindful eating, stress, and sleep quality.

## Delay Discounting for Substances of Use and Misuse

Substances with the potential for misuse are well-studied in the discounting literature, including various illicit drugs, alcohol, and cigarettes. Such substances are of interest to researchers given the various long-term negative consequences of their excessive consumption, including poor physical health, impacts on work or school performance, monetary constraint, and memory loss (McAlaney et al., 2021). Indeed, studying choices related to such substances within the DD paradigm is relevant given the use of a substance often includes the favoring of short-term reinforcers (i.e., effects of the drug) over the delayed consequences.

The literature linking substance abuse disorders to DD is robust and shows a number of trends. For individuals diagnosed with substance use disorders, the effect sizes for steep discounting are large in comparison with nonclinical samples (see meta-analysis by MacKillop et al., 2011). Although most of these discounting studies use monetary discounting, a number of others have used specific drugs as outcomes that are relevant to the substance use disorder studied. These nonmonetary drug outcomes, including opioids (Madden et al., 1997; Moses et al., 2020; Stoltman et al., 2015), alcohol (e.g., Petry, 2001), and nicotine (e.g., Johnson et al., 2007). Across studies, these drug-related outcomes are discounted hyperbolically.

In addition, there are compelling commodity-specific effects when drug values are equated and compared with money—drugs are consistently discounted more steeply than money. In a classic study by Madden et al. (1999), individuals with heroin dependence discounted monetary outcomes more steeply than nonusing controls, but heroin-dependent participants also discounted heroin more steeply than they did money. These findings were replicated in studies comparing DD for drug-related and monetary outcomes among individuals with cocaine use disorder (Bickel et al., 2011a, b) and with cannabis use disorder (Jarmolowicz et al., 2020; Lee et al., 2015). However, it is important to note that in the Jarmolowicz et al. study, the higher rates of discounting with cannabis were linked to differences in sensitivity to magnitude. Other studies also have found that magnitude sensitivity may contribute to commodity differences in rats (Locey & Dallery, 2009); therefore, it may be necessary to use mathematical discounting models that parse this variable from delay sensitivity.

Hyperbolic discounting has also been exhibited with licit drug outcomes such as alcohol. Petry (2001) compared DD for alcohol with individuals experiencing current alcohol use problems, individuals in recovery from alcohol use problems, and a control group of individuals who had never experienced alcohol use problems. Individuals with alcohol use problems exhibited steeper discounting for alcohol compared to controls, with notably steeper discounting among current drinkers. Moreover, alcohol was more steeply discounted than money. Likewise, Phung et al. (2019) evaluated DD for alcohol and money in a sample of individuals with alcohol use disorder (AUD) and found that individuals with AUD had steeper discounting for both money and alcohol than non-AUD participants, but those with AUD discounted alcohol more steeply than money.

Nicotine use also predicts steeper discounting processes, though most research shows this trend with monetary outcomes (Baker et al., 2003; Bickel et al., 1999; Reynolds et al., 2004; Reynolds & Fields, 2012; Stein et al., 2016). However, a number of studies examining DD for cigarettes have found that current smokers discount cigarettes more than ex-smokers and nonsmokers and discount cigarettes steeper than money (Baker et al., 2003; Bickel et al., 1999; Johnson et al., 2015a, b). Vaping nicotine appears similar. When monetary outcomes are used, current vapers discount money more steeply than nonvapers or former vapers (Weidberg et al., 2017), but current vapers also discount e-cigarette liquid more steeply than money (Pericot-Valverde et al., 2020).

In all instances, those with high valuation and experience with the drug (such as those who misuse licit and illicit drugs) show steeper discounting than nonusing controls. In addition, drug-related outcomes, whether illicit or licit, are discounted more steeply than money, supporting commodity-specific effects. Like food, drugs are considered primary reinforcers, and this property, along with its directly consumable nature and high perishability (Odum et al., 2020) may explain this commodity specificity.

It is noteworthy to point out that, similar to monetary discounting studies, most of the discounting research on drug-related outcomes use same-commodity choices. This allows the qualitative features of choices to be held constant so delay and amount of drug can be systematically manipulated. In the real world, however, this interpretation may be problematic. More drug later (the putative self-controlled choice) is a choice that actually places health at greater risk. To extend same-commodity research, cross-commodity discounting studies, in which participants choose between the drug of interest and a nondrug reinforcer—often money, have been conducted. Cross-commodity choices are important for establishing and understanding preferences for nondrug alternatives, which are often targets for treatment.

In one study with cross-commodity discounting (Bickel et al., 2011a, b), DD for money and cocaine with individuals with cocaine dependence was assessed in both same commodity (cocaine now vs. cocaine later [CC]; money now vs. money later [MM]) and cross commodity (cocaine now vs. money later [CM] and vice versa [MC]) formats. Discounting was strongest under the MC condition, followed by CC, CM, and MM. In other words, when cocaine was offered as the larger, later (LL) option, the steepest discounting was found; when money offered as the LL, discounting values were the lowest. This shows that when the drug is the delayed outcome, a nondrug outcome available immediately like money may induce greater discounting, in this case, preferences for the more immediate reinforcer. Moody et al. (2017) found similar alcohol commodity effects in non-clinical alcohol users—alcohol was discounted more steeply than money in same-commodity conditions, but with cross-commodity DD tasks, when alcohol was the delayed outcome, discounting was the steepest. These studies extend the literature on same-commodity studies by showing that discounting changes depending on what commodity is offered as the delayed outcome. These studies also enhance external validity by examining choices involving a nondrug alterative.

### Drug Delay Discounting and Treatment Specificity

Some studies that have used treatments to reduce drug use have also examined the effects of these treatments on monetary discounting. The majority of these studies report no drug treatment-related effects on monetary discounting (Aklin et al., 2009; Black & Rosen, 2011; Dennhardt et al., 2015; De Wilde et al., 2013; Littlefield et al., 2015) even when there are clinically relevant reductions in drug consumption. One exception was a study by Landes et al. (2012), who reported the effects of a multiple-component drug treatment study that reduced opioid use. Although this study reported a significant decrease in monetary discounting, these discounting reductions did not predict abstinence outcome. These studies occasion one to wonder if drug-related outcomes (especially those targeted in the treatments) were used in addition to monetary outcomes in the discounting tasks, whether stronger commodity-specific changes in discounting might be revealed or whether drug-specific discounting patterns would be more directly tied to abstinence outcomes.

Other treatment-based studies that reduce the consumption of drugs in individuals with chronic substance use or SUDs compare discounting across drug-related and monetary commodities and have found commodity-specific treatment effects. One study by Yoon et al. (2009), for example, showed that a contingency management (CM) program, in which participants receive vouchers for achieving nicotine reduction goals, reduces cigarette smoking. In addition, the CM program reduced DD for cigarettes (choices for immediate cigarettes over delayed ones) but did not affect monetary DD.

Likewise, Lee et al. (2015) used a multiple component treatment for cannabis use disorder to reduce cannabis use, but also examined discounting for money and cannabis as dependent variables. Their results showed that after treatment, discounting for cannabis and money decreased for both adolescents and adults. However, there were stronger effect sizes for the decreases in cannabis compared to money. Therefore, this treatment that reduced cannabis consumption also showed stronger commodity-specific effects in discounting that were related to the target drug.

Other SUD treatments with objectives to reduce drug consumption have also found that the treatment reduces both drug and money-related discounting. One study by Yi et al. (2008), for example, showed that an effective CM program for reducing cigarette consumption also reduced DD for cigarettes and for money. Although the effects were not specific to discounting for cigarettes, using both a monetary and nonmonetary outcome shows that the shifts in discounting that occur with this treatment occur with at least two commodities (and perhaps more), reflecting a general change in the behavioral process of discounting. Therefore, using more than one commodity and especially one related to the treatment (i.e., cigarettes) have benefits that go beyond only using monetary as an outcome for a smoking cessation treatment in terms of identifying specific or general changes in behavioral processes that may also manifest in other nondrug-based decisions, such as those with money.

### Summary

In sum, drugs are discounted hyperbolically and more steeply than money. Drugs are also more steeply discounted by individuals with drug reinforcer pathologies compared to controls who do not use drugs or who formerly use drugs. This commodity effect creates larger ranges in variation discounting, which also reduces the chances of ceiling effects in treatment studies. Indeed, treatments that are aimed to reduce drug consumption benefit by including DD tasks to evaluate preferences for drug. Those that include both drug and money outcomes tend to report commodity-specific effects related to the target drug whereas others may report cross-commodity effects that likely affect the general process of discounting.

## Delay Discounting for Sexual Outcomes

Sex is another nonmonetary outcome that has been studied within the discounting framework (see Johnson et al., 2021, for review). As important as DD may be to the understanding of sexual decisions, assessing the value of something as subjective and diverse as sex is both complicated and nuanced in comparison to money and other nonmonetary commodities. Researchers have used various methods to characterize DD-related sexual choices. In the first study to apply DD to sexual outcomes (Lawyer, 2008), participants chose between smaller, sooner amounts versus larger-later amounts of their favorite type of erotica (e.g., 10 min now vs. 30 min in 3 hr). Results showed that individuals who typically view erotica are more likely to show the prototypical hyperbolic DD pattern for sexual outcomes. Those who do not view or value erotica, however, did not show this pattern, even though both groups displayed hyperbolic discounting for money. Lawyer et al. (2010) conducted a similar study to broaden sexual DD decisions beyond erotica by asking sexually active participants to make DD choices regarding hypothetical sexual activity, which has more relevance to dyadic sexual acts than choices regarding erotica. The results from this study also showed that sexual discounting could be described using the hyperbolic decay model, supporting the use of sexual activity as an outcome within the DD paradigm.

Commodity-specific DD effects have also been examined between sexual and monetary outcomes. For instance, Jarmolowicz et al. (2014a, b) reported on sexual and monetary discounting in individuals who use cocaine, a segment of the population known to have steeper discounting patterns in general (e.g., Heil et al., 2006). Participants first equated a specific number of sexual acts to $1,000, which allowed researchers to directly compare money to sex. Sexual discounting in this sample was steeper than monetary discounting for both men and women.

In addition, Holt et al. (2014) compared sexual, monetary, and food-related outcomes in college students. Here, sexual outcomes were characterized using quality (the “ideal” sexual experience) rather than duration or number of sex acts. Participants chose between receiving their full-length “ideal” sexual experience after a delay, or to receive a slightly less than “ideal” sexual experience now. Sexual discounting was steeper than money discounting. In addition, the study replicated that food is more steeply discounted than money, but food was also more steeply discounted than sex. In other words, food was more steeply discounted than sex, which was more steeply discounted than money.

Some studies have indirectly examined commodity-specific effects with sex compared to money in ways other than equating their value; they examine the degree to which each type of outcome is independently predicted by clinically relevant variables. For example, Lawyer and Schoepflin (2013) found greater discounting of sexual activity, but not monetary outcomes, was significantly related to self-reported sexual excitability. In addition, sexual discounting was unrelated to nonsexual outcomes or sexual inhibition. It is important to note that monetary discounting was unrelated to sexual excitability or sexual outcomes, showing that measures of sexual excitement are specifically predicted by discounting for sexual outcomes. In addition, Mahoney and Lawyer (2018) found that discounting for sexual activity was associated with the subscale of the Delaying Gratification Inventory specifically related to the domain of physically pleasurable events. Discounting for money, however, was not associated with this subscale. These two studies, then, show commodity-specific effects in which sex discounting, but not money discounting, predict measures of sexual arousal and excitation.

### Sexual Delay Discounting and Sexual Risk Behaviors

The context in which one participates in sexual behaviors could be considered “risky” when it increases the chance of one’s exposure to a negative outcome (Chawla & Sarkar, 2019). Individuals participating in sexual activities that can be considered risky are often choosing immediate reinforcers (i.e., unprotected sex) that have long-term negative consequences that are not as potent drivers of behavior due to their temporal distance from the act itself. Though sexual risk behaviors have not yet been considered within the framework of reinforcer pathologies, it may make sense to do so.

Lawyer et al. (2010) posited that discounting for sexual activity provides an opportunity to better understand such real-world sexual risk behaviors, and more important, is more meaningful when examining sexual risk than discounting for other commodities such as money. Indeed, sexual risk taking and sexual discounting are positively related (Sweeney et al., 2020). Lawyer and Mahoney (2017) also showed that DD (and probability discounting) for sexual outcomes were both differentially related to a self-report measure of sexually risky behaviors.

Condom use is an important risk behavior related to sexual health and reduces the risk of sexually transmitted infections (STIs), including HIV and viral hepatitis, as well as unwanted pregnancy (CDC, 2023c). Johnson and Bruner (2012) developed a discounting procedure for risky sexual behavior and condom use called the Sexual Discounting Task (SDT). They asked participants to indicate the likelihood of having immediate unprotected sex (i.e., without a condom right now) or delayed protected sex (i.e., with a condom in 3 hr) with specific photographed individuals judged to be the most/least sexually desirable or most/least likely to have an STI. They found that participants demonstrated significantly greater discounting (i.e., preference for unprotected sex immediately) for partners considered to be the most sexually desirable or least likely to have an STI versus those found least sexually attractive or most likely to have an STI, demonstrating a pattern of devaluing delayed condom-protected sex. This pattern was specific for sexual discounting and did not extend to monetary discounting.

Other studies using the SDT show that self-reporting of having sex when a condom is not available predicts steeper DD for unprotected sex (Sweeney et al., 2020). This risky pattern for unprotected sex has also been found in sexually diverse populations. For instance, in men who have sex with men, unprotected anal intercourse predicts steeper sexual discounting using the SDT (Herrmann et al., 2015). This effect was also specific to sexual discounting, as monetary discounting showed no relation with unprotected sex (Jones et al., 2018).

Other clinically relevant phenomena predict steeper sexual discounting. Substance use disorders, for instance, are associated with sexually risky behavior and sexual discounting. For instance, women with opioid dependence more steeply discount condom-protected sex compared to women without opioid dependence (Herrmann et al., 2014). In addition, survivors of sexual trauma tend to have higher rates of sexual risk behaviors (see review by Holcomb et al., 2021). Research with sexual discounting shows that sexual trauma predicts not only sexual risk taking, but also steeper rates of sexual DD (Mahoney et al., 2022). Therefore, SUDs and sexual trauma history are associated with greater sexual discounting.

### Sexual Discounting and Treatment Specificity

Discounting of delayed sexual outcomes could aid in the identification of high-risk individuals as well as serve as an important dependent variable for treatments that reduce sexually risky behavior (Johnson et al., 2021). No studies to our knowledge have assessed the degree to which treatments to reduce risky sexual behavior also reduce sexual discounting or monetary discounting for that matter. Researchers may wish to examine these areas in the future.

### Summary

In sum, sex is an important primary reinforcer. Because of its nuanced nature, it has received less attention in the discounting literature. Nonetheless, sexual outcomes in discounting have been quantified in a number of ways, including erotica and imagined sexual activity. Like food and drugs, sexual outcomes are discounted consistently more steeply than money, demonstrating commodity effects. Sexual discounting is also consistently associated with sexual risk behaviors, including condom nonuse; money discounting appears unrelated to sexual risk.

## Basic Experimental Properties of Nonmonetary Outcomes

The trends in commodity-specific effects with nonmonetary outcomes, as well as their consistent associations with reinforcer pathologies and risky health behaviors, supports their external validity in discounting research. However, the use of nonmonetary outcomes also requires other basic “litmus tests” to assure researchers of other properties of experimental rigor. One way to enhance confidence is to compare discounting findings with nonmonetary outcomes to established trends with monetary outcomes. There are a number of replicable phenomena in the literature with monetary discounting that can serve as a basis for other forms of validation of nonmonetary outcomes. These include the extent to which nonmonetary outcomes have magnitude effects, strong test–retest reliability, the similarity between real and hypothetical outcomes, and predictable age-related effects. These trends are summarized here.

### Magnitude Effects

Higher magnitude amounts of money tend to be consistently discounted less steeply than lower amounts of money—a phenomenon called the magnitude effect. This is the case whether using computerized discounting tasks (e.g., Green et al., 1999; Green et al., 2013) or the Monetary Choice Questionnaire (e.g., Kirby & Maraković, 1996; Hendrickson et al., 2015). Thus, one question concerns whether there are magnitude effects with nonmonetary outcomes, and if the trend is similar, i.e., smaller amounts are the most steeply discounted. Indeed, magnitude effects with hypothetical food have been reported in the literature (Hendrickson et al., 2015; Hendrickson & Rasmussen 2017; Odum & Rainaud, 2003; Rodriguez et al., 2021). For instance, small amounts of hypothetical pizza ($10 worth), for example, are discounted more steeply than large amounts of pizza ($100 worth; Odum et al., 2006). The Food Choice Questionnaire (Hendrickson et al., 2015), patterned after the Monetary Choice Questionnaire (Kirby & Maraković, 1999), also shows replicable magnitude effects with amounts of food and delays that are lower and representative of single sittings (up to 10 hr as opposed to months or years (Hendrickson et al., 2015; Hendrickson & Rasmussen 2017; Rodriguez et al., 2021). Magnitude effects have also been reported with drugs such as cannabis (Lee et al., 2015) and cigarettes (Johnson et al., 2007). Across these studies, smaller amounts of the nonmonetary outcome were discounted more steeply than larger amounts. To date, there have been no studies that report magnitude effects with sexual outcomes.

### Test–retest Reliability

The degree to which an individual’s rate of discounting is correlated at two different time points refers to test–retest reliability and indicates not only the replicability of the process across time as a research tool, but also its stability across time as a trait-like behavior (Odum, 2011a,1b). Measures of monetary discounting, including both titration and short form measures, have had relatively extensive research into their reliability Tasks of monetary discounting such as the Monetary Choice Questionnaire (MCQ; Kirby & Maraković, 1996) have successfully tested reliability. When discounting is tested even 1-year later, reliability of monetary discounting is strong and on par with reliability of personality traits (Kirby, 2009).

Test–retest reliability in discounting has also been examined with food. The FCQ has generated strong reliability, and statistical equivalence, across three time points separated by weeks (Musquez & Rasmussen et al., 2024). In addition, in the control arms of randomized control studies with mindful eating and food DD, discounting for food was highly correlated across two to three sessions with 1 week between sessions (Hendrickson & Rasmussen, 2013, 2017; Rasmussen et al., 2022). Food discounting also appear statistically similar consistent across measures, e.g., adjusting amount versus choice questionnaire (Hendrickson et al., 2015) Therefore, food discounting is not only reliable within sessions, but also valid across other measures.

To date, only one study has examined test–retest reliability with sexual outcomes. Johnson and Bruner (2013) tested reliability across 1-week periods using the Sexual Discounting Task in a sample of participants with cocaine dependence. They reported statistical equivalence across four different conditions (most and least likely to have and STI and most and least want to have sex with). No research to date to our knowledge has examined the test–retest reliability of DD for drugs or alcohol. As such, more research is needed regarding this property of commodity-specific DD measures.

### Hypothetical versus Real Outcomes

A growing literature compares real (or potentially real) versus hypothetical monetary outcomes in DD studies. These studies consistently show that hypothetical and real (or potentially real) outcomes are discounted similarly (Lawyer et al., 2011; Lagorio & Madden, 2005; Madden et al., 2003; Madden et al., 2004; Robertson & Rasmussen, 2018) and even statistically equivalent (Matusiewicz et al., 2013). Some studies also compare potentially real versus hypothetical nonmonetary outcomes. Robertson and Rasmussen (2018), for instance, compared potentially real food discounting to hypothetical food discounting and found them to be highly correlated and statistically equivalent. These correlations were also found in a Czech sample (Rasmussen et al., 2021). It is important to note that these studies support the trend of similarity in real versus hypothetical rewards, further validating their use.

Fewer studies compare outcomes related to drugs and sexual outcomes and this is likely because of the ethics involved in delivering the real outcomes. One exception, however, compared potentially real cigarettes to hypothetical cigarettes in smokers and found real cigarettes were discounted more steeply than hypothetical cigarettes (Green & Lawyer, 2014), thereby showing that using cigarettes as outcomes in discounting may be more nuanced than other outcomes. More research on real versus hypothetical outcomes with drug and sexual stimuli is needed, though ethical delivery of these types of outcomes should be strongly considered.

### Predictive Effects of Age

Age has been shown to be inversely related to monetary discounting (Green et al., 1994, 1996, 1999; Steinberg et al., 2009). In other words, as age increases, discounting for money decreases. One exception published by Read and Read (2004) showed that monetary discounting is more U-shaped with the steepest discounting occurring in childhood and older age. Fewer studies have characterized the relation between age and discounting with nonmonetary outcomes, but age-related effects with food-related outcomes have been shown. Lee and Rasmussen (2021) showed that food discounting across the lifespan changes in a U-shaped manner. From early childhood to adolescence, discounting for food decreases with age. Once adulthood is reached, however, discounting for food increases from early adulthood to later adulthood, which corresponds to changes in metabolism that increase BMI.

To date, there are no studies to our knowledge that examine the relation between age and other nonmonetary outcomes related drugs and sexual outcomes. However, there are established trends in how the reinforcing efficacy of drugs and sex may change with age. For instance, the prevalence of substance use disorders peaks in early adulthood, and then decreases with age (Heyman, 2009; Schulte & Hser, 2013; National Institute on Drug Abuse [NIDA], 2023), though middle-aged and older adults may still develop substance use disorders (e.g., see NIDA, 2023; Stewart et al., 2022). This trend may indicate that discounting for drugs (and not just money) may also decline with age. For those who develop an SUD later in life, it could be useful to identify factors that increase this likelihood and whether discounting processes are related.

Regarding sexual outcomes, some research suggests that for men and women, physiological and psychological changes that accompany transitions to middle and older adulthood may decrease sexual arousal and functioning (e.g., Araujo et al., 2004; Purifoy et al., 1992; Thompson, et al., 2011), though sexual interest and enjoyment may also persist well into later adulthood, especially when health is satisfactory (e.g., Thompson et al., 2011; Traeen et al., 2018). Complicating the picture, in older adults, sexual risk taking, such as a heightened prevalence of sexually transmitted infections, increases (Johnson, 2013) and this risk is greater for men who have sex with men (Poynton et al., 2013). It should also be noted that less is known about other genders and sexualities (e.g., transgender, pansexual) in middle-aged and older adults. Therefore, it would be important to characterize how DD for sexual outcomes, as well as drug outcomes, changes across the lifespan for all genders, and the extent to which health-related variables may coincide with discounting processes.

## Limitations of Nonmonetary Outcomes in Discounting Research

### Value and Variation in Preferences of Nonmonetary Outcomes

In discounting, individual variation in the value of food, drugs, and sexual outcomes creates challenges for measurement compared to monetary outcomes. At one extreme are reinforcer pathologies, in which a specific outcome is valued to the detriment of other long-term outcomes, such as good health. But even with most people that do not have reinforcer pathologies, there is wide variation in preferences. To use one food type, such as marshmallows, cookies, or pizza in a food DD task, for instance, assumes that all participants value pizza and marshmallows in the same manner. One way in which researchers have gotten around this problem is to use imagined standardized bites of favorite foods (e.g., Rasmussen et al., 2010). The same is true for sexual outcomes in which minutes of ideal individualized sexual experiences are used as the outcome (Lawyer et al., 2010; see Johnson et al., 2001, for review).

On the other extreme are individuals who do not value the nonmonetary outcome at all. For instance, we have found informally (though, not published) that approximately 6%–10% of individuals (usually highly restrained eaters or very thin individuals) with food discounting tasks prefer a smaller amount of food over a larger. In some instances, food discounting patterns are more random. When individuals do not value more of an outcome, it confounds the interpretation of choices for the delayed outcome. For example, Lawyer (2008) found that, for some participants who show low interest in erotica (or view it negatively), patterns of DD could not be described using the hyperbolic decay mode. It is important, then, for research to include a discounting question that assesses preference for quantity first as a screener and establish a priori rules for inclusion of data.

### Nonsystematic Data

Money may be a frequently used outcome because participants respond systematically to it. Smith et al. (2018) conducted a meta-analysis on the percent of systematic responding across monetary and nonmonetary commodities using Johnson and Bickel’s (2008) algorithm for determining nonsystematic data. Across 114 studies, about 20% of discounting data yield nonsystematic response patterns, and some of this may be due to random responding (see Craft et al., 2022). A disproportionately higher percent of those patterns, however, occur with nonmonetary outcomes. Explanations may include individual valuation, though state-based factors, such as deprivation, might differentially influence discounting for nonmonetary commodities.

### Deprivation

Deprivation, an establishing operation (EO) that momentarily increases the potency of a reinforcer and the response probability on which it is contingent (e.g., Laraway et al., 2003; Tapper, 2005), is a variable that is often controlled in operant procedures that investigate reinforcer efficacy. Deprivation may also affect discounting processes. With monetary outcomes, it is difficult (if not impossible) to deprive a participant of their own money, so researchers have developed other methods of investigating this EO, including comparing participants with lower versus higher income. Those with lower income discount money more steeply than those of higher income (e.g., Rodriguez et al., 2021). Other more experimental studies manipulate narratives in which there is a loss of financial status or resources; these studies show increases in discounting for money during the “financial deprivation” conditions (Callan et al., 2011; Moeini-Jazani et al., 2019). Therefore, across both types of monetary deprivation studies appear to enhance preferences for immediate monetary outcomes.

Given that the types of outcomes that reliably predict reinforcer pathologies are primary reinforcers, such as food and drugs, and these have historically been affected by EOs such as deprivation (Michael, 1993; Tapper, 2005), it would be critical to understand the degree to which deprivation reliability predicts discounting with these types of outcomes. Once understood, they too, can be controlled either methodologically, statistically, or both.

### Food

Human discounting studies with real food show that aspects of deprivation may enhance preferences for smaller, more immediate food outcomes. For instance, Logue and King (1997) showed that individuals who were currently dieting preferred smaller, sooner juice compared to waiting for larger, delayed amounts of juice after a delay. Though deprivation was not systematically manipulated, this suggests that dieting status, may affect food discounting. In a more systematic way, Kirk and Logue (1997) showed that the opposing process of satiation decreases discounting for food. Here, consuming a soup pre-load before a discounting task with SS versus LL apple juice amounts reduced discounting for apple juice compared to no preload.

Others studies have systematically evaluated the effects of food deprivation on hypothetical food outcomes by experimentally inducing a fast with human participants. Skrynka and Vincent (2019), for example induced a 10-hr versus 1-hr fast (validated with by blood-glucose levels) and examined discounting for hypothetical chocolate, money, and music. They found that all three commodities were discounted more steeply after the fast, but the largest effects were found for chocolate, suggesting stronger commodity-specific effects. Some researchers have attempted to control deprivation by implementing a 2–4 hr fast before a session, verifying the self-report with a blood glucose test (e.g., Law & Rasmussen, 2023; Lee & Rasmussen, 2021; Rasmussen et al., 2022; Rodriguez et al., 2021). In these studies, deprivation was unrelated to food discounting, but subjective hunger predicted steeper food discounting, though not monetary discounting.

It is noteworthy that the more systematic effects of deprivation on discounting with real food have also been examined with animal studies, which removes the subjectivity of visceral interoreceptive stimuli. In an article by Oliviera et al. (2013), for example, pigeons underwent two types of deprivation (maintaining weights at 75%–80% versus 90%–95% of their free-feed weight and 23 hr versus 1 hr of food deprivation); neither type of deprivation affected food discounting. Therefore, the data on food deprivation affecting food discounting are somewhat mixed.

### Drugs

Research on the effects of deprivation with discounting for drugs has been conducted with individuals who chronically consume drugs or are diagnosed with SUDs. In some samples, the individuals experience withdrawal symptoms during deprivation. For example, Giordano et al. (2002) showed that for individuals being treated with buprenorphine for a current opioid SUD, mild opioid withdrawal enhanced DD for heroin (and money), but was attenuated with a buprenorphine dose. In experiments that use hypothetical deprivation, in which narratives instruct those who currently use heroin to imagine deprivation and withdrawal effects, discounting for hypothetical heroin as an outcome increased compared to a no-deprivation condition (Moses et al., 2019; Stoltman et al., 2015).

The effects of nicotine deprivation have also been examined with smoking. In a within-subjects study with smokers (Field et al., 2006), a 13-hr nicotine deprivation versus smoking as usual was induced. Steeper discounting occurred with deprivation for both cigarettes and money compared to no deprivation. Yi and Landes (2012), however, showed that 24 hr of nicotine deprivation compared to smoking as normal resulted in increases in monetary discounting but not DD for cigarettes. Studies with electronic nicotine delivery systems also show that nicotine deprivation (16 hr) increases preference for smaller–sooner e-liquid options when larger–later monetary outcomes were available, however deprivation did not affect discounting when both options were money or e-liquid (Pericot-Valverde et al., 2023). This preference for cigarettes over money was also shown with 24 hr of nicotine deprivation in a study by Mitchell (2004), though nicotine deprivation did not affect discounting when the choices were between money.

### Cross-Commodity Deprivation Effects

Some studies have examined the effects of deprivation of one outcome on a different type of outcome or commodity. Skrynka and Vincent (2019), for example, found that food deprivation induces steeper discounting for not only food, but also money and music. Another study showed that skipping breakfast increases money discounting (Bartholdy et al., 2016). Therefore, food deprivation may increase discounting for outcomes other than food. Other studies with smokers have induced an 18–24-hr deprivation of nicotine and tested the effects on monetary discounting and found no effects of deprivation on monetary discounting (Ashare & Kable, 2015; Ashare & McKee, 2012; Miglin et al., 2017; Mitchell, 2004; Roewer et al., 2015; Yoon et al., 2009). Still, others report significant, though small, effect sizes of nicotine deprivation on monetary discounting (Heckman et al., 2017). Some studies, however, report stronger effects. Using nicotine deprivation levels that varied from 8 hr to 24 hr, hypothetical money discounting increased compared to nondeprived control conditions (Ashare & Hawk, 2012; Grabski et al., 2020; Yi & Landes, 2012).

Overall, then, the effects of deprivation on discounting for food and drug are mixed (see also meta-analysis by Downey et al., 2022). Across a number of contexts with humans (and animals), deprivation in and of itself does not necessarily or consistently affect discounting processes. One trend, however, is the extent to which visceral or interoceptive feedback may be salient enough with a deprivation condition to reach a threshold for detection, which may also be related to conditions of food and drug withdrawal (Downey et al., 2022). It is important also to note that when details of deprivation are verbally described in narrative manipulations or when scales of subjective hunger (which ask participants to attend to interoceptive cues) are used, deprivation effects are more likely to be detected. Therefore, it may be important to more systematically examine the visceral (i.e., interoreceptive and verbal) aspects of deprivation to better understand the impact on discounting with humans. Finally, it is important to note that no studies to date have examined the effects of deprivation on sexual discounting. Although sexual outcomes are nuanced, deprivation may also vary in terms of individualized duration. Future research should attempt to characterize these effects.

## Discussion and Future Directions

The research to date shows compellingly that examining DD for nonmonetary outcomes is critically important to the discounting literature. Primary reinforcers such as food, drugs, and sexual outcomes are not only ecologically valid, but they are also directly relevant to choices that are implicated in health and reinforcer pathologies such as substance use disorders, obesity, and sexual risk behavior. Food, drugs, and sexual outcomes are consistently discounted more steeply than monetary outcomes, which creates larger windows of variation to evaluate treatment effects. Indeed, treatments aimed at reducing health-related problems with these reinforcers are often more effective when nonmonetary outcomes are used instead of, or with, monetary reinforcers.

Like money, DD for food-related outcomes shows strong test–retest reliability, magnitude effects, statistical equivalence for real versus hypothetical outcomes, and predictable age-related effects. Limited research on drug-related outcomes shows some evidence for magnitude effects. DD for sexual outcomes has strong test–retest reliability but have not yet been validated on other properties. Future research should focus on characterizing more of these methodological properties, especially with drug and sexual outcomes. Deprivation effects on DD for food and drugs (as well as money) as outcomes seem to be mixed, but more visceral and narrative description of these variables seem to be better predictors of discounting than deprivation. More research on these aspects is needed, in addition to more methodological studies with sexual outcomes. We also recommend that researchers control for deprivation when using nonmonetary outcomes, or at least include measures of actual and subjective deprivation, such that they can be controlled.

The limitations with nonmonetary reinforcers unsurprisingly include factors related to individual preferences. Variation in discounting for specific outcomes is supported by data that shows personal valuation and hedonic preferences, including those from reinforcer pathologies. DD for nonmonetary outcomes also have more variation in nonsystematic data (Smith et al., 2018), and this likely comes from preferences as well. This is a lesser concern with monetary discounting which is consistently more valued across participants and therefore yields more systematic data. This issue, incidentally, is not different from most behavior analytic research using reinforcers. The consensus in the field has always assumed that preferred outcomes, i.e., reinforcers, are individualized. Researchers simply need to find ways to standardize, control, or leverage those differences. The use of bites of a favorite food or minutes of a preferred sexual experience are relatively easy ways to do this with food and sexual outcomes, respectively.

We strongly believe that the benefits of using nonmonetary outcomes outweigh the limitations, especially when the limitations can be methodologically and statistically controlled. Therefore, we recommend that researchers use both monetary and nonmonetary outcomes in their research. This suggestion is especially the case if studies involve health choices, special populations that may have reinforcer pathologies, or consumption of specific nonmonetary reinforcers. Using both outcome types can also assist in evaluating commodity specific effects. In addition, we recommend more studies on cross-commodity discounting in which a commodity besides the nonmonetary or opt-out option is used. This also creates greater ecological validity.

We have additional recommendations that are clinical and health related in nature. To date, though discounting processes predict reinforcer pathologies and range of clinical symptoms, there are no normative data that may predict at-risk behavior. Researchers, especially those in clinical fields, may wish to collect data on discounting when conducting clinical intakes, such that normative data may be generated and cut-off criteria for potential pathological behavior may be identified. Collecting data across commodities would be especially helpful, especially in terms of examining cross-commodity discounting patterns or specific commodities that may predict certain health or mental health problems more readily than others. These normative data may also help define what a “high *k*” or steep discounter actually means in terms of risk.

A final recommendation is to conduct research that optimizes and standardizes methods for measuring DD, especially in special populations. Adjusting-amount or adjusting-delay procedures may take up to 30 min to administer, whereas 27-item choice-questionnaire formats (i.e., Hendrickson et al., 2015; Kirby & Maraković, 1996) or briefer titration procedures are quicker to administer (5 min). Indeed, shorter, but valid, instruments are an important concern for clinical intakes or working with vulnerable populations in the community. Monetary and food discounting have been compared and validated across adjusting-amount procedures versus choice questionnaire formats and found to generate similar discounting values with smokers and college students (Epstein et al., 2003; Hendrickson et al., 2015). However, more research is needed with these outcomes, and with sexual outcomes, including with special populations to determine the extent to which these measures generalize.
